# 

**Published:** 1864-07

**Authors:** 


### BOOK NOTICES AND REVIEWS.

### *Books and Pamphlets Received.—*

*The Principles and Practice of Obstetrics:* Illustrated with One Hundred and Fifty-Nine Lithographic Figures from Original Photographs and with numerous Wood Cuts By Hugh L. Dodge, M. D., Emeritus Professor of Obstetrics and Diseases of Women and Children, in the University of Pennsylvania; lately one of the Physicians to the Lying in Department of the Pennsylvania Hospital; lately one of the Physicians to the Philadelphia Almshouse Hospital; Consulting Physician to the Philadelphia Dispensary; Fellow of the College of Physicians of Philadelphia; Member of the American Philosophical Society, etc., Author of a treatise on "The Peculiar Diseases of Women." Philadelphia: Blanchard & Lea. 1864. From S. C. Griggs & Co., No. 39 and 41 Lake street, Chicago.

*The Pathology and Treatment of Venereal Diseases Including the Results of Recent Investigations upon the Subject* By Freeman J. Bumstead, M. D., Lecturer on Venereal Diseases at the College of Physicians and Surgeons, New York; late Surgeon to St. Luke' Hospital; Surgeon to the New York Eye and Ear Infirmary. A New and Revised Edition, with Illustrations. Philadelphia: Blanchard & Lea—1864. From W B Keen, Chicago.

*A Manual of the Practice of Medicine.* By Thomas Hawkes Tanner, M. D., F. L. S., Member of the Royal College of Physicians; Assistant Physician for the Diseases of Women and Children to King's College Hospital etc., etc From the last London Edition, Enlarged and Improved. Philadelphia: Lindsay & Blakiston—1864. From S. C, Griggs & Co.

*On Rheumatism, Rheumatic Gout, and Sciatica, their Pathology, Symptoms and Treatment* By Henry William Fuller, M. D., Cantab. Fellow of the Royal College of Physicians, London; Physician to St. George's Hospital, etc., etc. From the last London Edition. Philadelphia: Lindsay & Blakiston —1864. From S. C. Griggs & Co,

*A Treatise on the Chronic Inflammation and Displacements of the Unimpregnated Uterus.* By Wm. H. Byford, A. M. M. D., Professor of Obstetrics, etc., etc., Chicago Medical College, Medical Department Lind University. Philadelphia: Lindsay & Blakiston—1864. From S. C. Griggs & Co.

*A Practical Treatise on the Sexual Organs of Women.* By F. W. Von Scanzoni. Translated from the French by Drs. H. Dor and A. Soci[illegible], and Annotated, with the Approval of the Author, by Augustus K. Gardiner. A. M. M. D. With upwards of Sixty Illustrations. R. M DeWitt, Publisher, 13 Frankfort street, New York. Through John R. Walsh, Chicago.

*Transactions of the State Medical Society of Indiana,* at the Fourteenth Annual Meeting, Held in the city of Indianapolis, May 1[illegible]th and 18th, 1864.

☞ Business letters to the JOURNAL should be addressed to Drs. MILLER & INGALS, Chicago, Ills., P. O. Drawer 5787. When money is received a receipt will be returned with the next number of the JOURNAL, and subscribers failing to get such receipt will confer a favor by giving us notice of any omission.

